# Electricity, Computing Hardware, and Internet Infrastructures in Health Facilities in Sierra Leone: Field Mapping Study

**DOI:** 10.2196/30040

**Published:** 2022-02-03

**Authors:** Emeka Chukwu, Lalit Garg, Edward Foday, Abdul Konomanyi, Royston Wright, Francis Smart

**Affiliations:** 1 Department of Computer Information Systems Faculty of Information Communications Technology University of Malta Msida Malta; 2 Directorate of Policy, Planning, and Information Ministry of Health and Sanitation Freetown Sierra Leone; 3 Directorate of eGovernment Ministry of Information and Communication Freetown Sierra Leone; 4 Monitoring and Evaluation Unit, Health and Nutrition United Nations Children's Fund Freetown Sierra Leone

**Keywords:** digital health, mHealth, eHealth infrastructure, health ICT, SpaceX, connectivity, Sierra Leone, rural-urban divide, rural areas, internet

## Abstract

**Background:**

Years of health information system investment in many countries have facilitated service delivery, surveillance, reporting, and monitoring. Electricity, computing hardware, and internet networks are vital for health facility–based information systems. Availability of these infrastructures at health facilities is crucial for achieving national digital health visions.

**Objective:**

The aim of this study was to gain insight into the state of computing hardware, electricity, and connectivity infrastructure at health facilities in Sierra Leone using a representative sample.

**Methods:**

Stratified sampling of 72 (out of 1284) health facilities distributed in all districts of Sierra Leone was performed, factoring in the rural-urban divide, digital health activity, health facility type, and health facility ownership. Enumerators visited each health facility over a 2-week period.

**Results:**

Among the 72 surveyed health facilities, 59 (82%) do not have institutionally provided internet. Among the 15 Maternal and Child Health Posts, as a type of primary health care unit (PHU), 9 (60%) use solar energy as their only electricity source and the other 6 (40%) have no electricity source. Similarly, among the 13 hospitals, 5 (38%) use a generator as a primary electricity source. All hospitals have at least one functional computer, although only 7 of the 13 hospitals have four or more functional computers. Similarly, only 2 of the 59 (3%) PHUs have one computer each, and 37 (63%) of the PHUs have one tablet device each. We consider this health care computing infrastructure mapping to be representative with a 95% confidence level within an 11% margin of error. Two-thirds of the PHUs have only alternate solar electricity, only 10 of the 72 surveyed health facilities have functional official internet, and most use suboptimal computing hardware. Overall, 43% of the surveyed health facilities believe that inadequate electricity is the biggest threat to digitization. Similarly, 16 (22%) of the 72 respondents stated that device theft is a primary hindrance to digitization.

**Conclusions:**

Electricity provision for off-electricity-grid health facilities using alternative and renewable energy sources is emerging. The current trend where GSM (Global System for Mobile Communication) service providers provide the internet to all health facilities may change to other promising alternatives. This study provides evidence of the critical infrastructure gaps in health facilities in Sierra Leone.

## Introduction

### Background

Globally, health systems technology infrastructure has been a topic of constant debate. Technology infrastructure can help fast-track attainment of the global Sustainable Development Goal targets. In 2004, the European eHealth Action Plan considered technology infrastructure as critical to deployed solutions [1]. Liu et al [2] explored the challenges and solutions of deploying eHealth infrastructure. Electricity was listed as one of the essential amenities by the World Health Organization (WHO) handbook on monitoring building blocks of the health system [3]. Omotosho et al [4] surveyed the current state of information and communications technology (ICT) and related infrastructure supporting eHealth deployment in Africa. However, their desk survey covered computing hardware but not electricity. Sierra Leone has approximately 100 megawatts of electricity installed, with electricity per capita estimated at 34 kilowatt hours [5,6]. As of 2019, an estimated 22.7% of Sierra Leoneans have access to electricity, with only 1.5% of those in rural areas having electricity access [5,6]. There are three leading mobile service providers in Sierra Leone and broadband internet utilization remains low [7].

Sierra Leone has 1284 health facilities, including 24 district hospitals spread across the 13 health care districts [8]. The health systems are split between Primary Healthcare Units (PHUs) and hospitals. The PHUs are classified into Community Health Centers (CHCs), Community Health Posts (CHPs), and Maternal and Child Health Posts (MCHPs) [9]. The MCHPs operate at the village level, which serve less than 5000 people and are mainly staffed by Maternal and Child Health (MCH) aides who provide mainly maternal health services [10]. The CHPs operate at the town level and are situated to serve 5000 to 10,000 people. The CHPs are staffed by a community health nurse and MCH aides. In addition to services provided by MCH, they also provide disease prevention and control. The CHCs cover the chiefdoms, with an estimated population of 10,000 to 20,000 people. The CHCs are staffed by Community Health Officers and those found in CHPs. The CHCs conduct disease surveillance services. Hospitals are located to meet service needs, with at least one per district. Health facilities can be either public or privately owned, although the majority are public. Investments in health information systems (HISs) has resulted in regular health facility service delivery and disease surveillance reporting, using the District Health Information System in recent years [8,11]. Other HIS data sources include health surveys, birth registrations, census, and health resource tracking (eg, health accounts) [3].

Approximately 98% of health facilities consistently submit aggregated service delivery data to the central repository [9]. In 2017, the Directorate of Planning, Policy, and Information (DPPI) at the Ministry of Health and Sanitation (MoHS) inaugurated the eHealth coordination hub to govern the systematic application of digital health solutions for health systems improvement through data [12]. This culminated in the launch of the first national digital health strategy 2018-2023 [9]. The vision of the national digital health strategy is to guarantee universal health coverage using ICT. According to this strategy, service delivery data and disease surveillance data are collected and aggregated using a mixture of paper and digital health tools. This health information flows from the community, PHUs, or hospitals up to the district. Universal health coverage is one strategy that can ensure meeting global health care targets for different health domains.

### Study Objective

The eHealth coordination hub commissioned a mapping of the digital health–enabling environment components in Sierra Leone’s health facilities in January 2019. This study unearths the state of digital health infrastructure as defined by the WHO-International Telecommunication Union eHealth strategy development toolkit [13] using a representative sample. The infrastructure (or information structures) that support collection, processing, and knowledge-mining of individualized patient data can be categorized as connectivity, computing hardware, and electricity. This study does not discuss other architectural (nonphysical) information structures such as standards and interoperability components. Instead, this study builds upon previous success to provide evidence of the linkage between the availability of these infrastructures and the availability of individualized digital health data in support of the national vision.

## Methods

### Health Facilities Sampling Strategy

**Figure 1:**
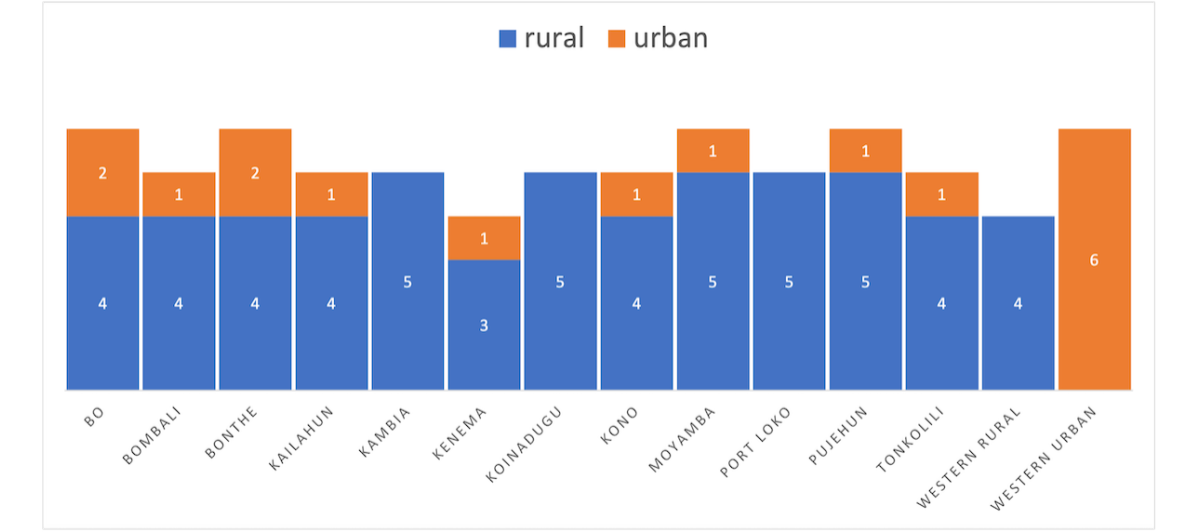
Distribution of surveyed health facilities by all districts and economic characteristics.

We used a stratified sampling strategy where each of the 13 health care districts in Sierra Leone was purposefully targeted for 5 or more health facilities. In total, 72 of the 1284 health facilities in Sierra Leone were selected for this mapping exercise, including 17 urban and 55 rural health facilities (Figure 1).

**Figure 2:**
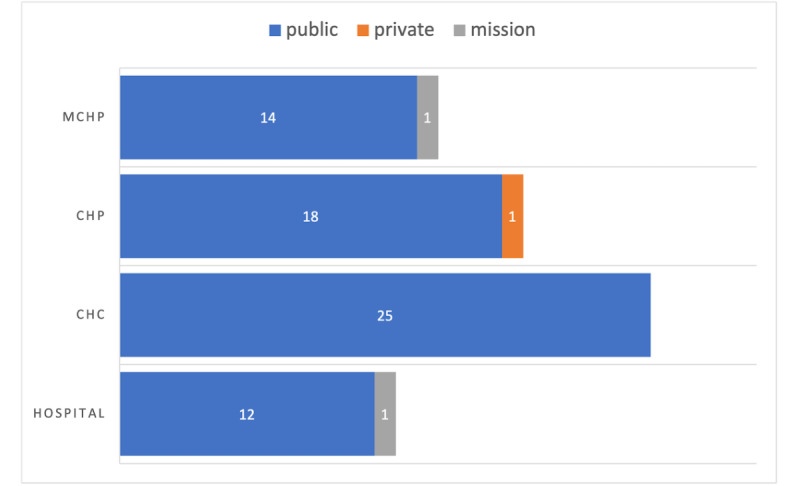
Health facilities surveyed, by ownership and type. MCHP: Maternal and Child Health Post; CHP: Community Health Post; CHC: Community Health Center.

The margin of error for the 72 samples was calculated to be 11% to yield 95% confidence that the sample is representative [14]. The health facilities surveyed and their distribution, by ownership and type, are given in Figure 2. Among the 72 health facilities sampled, 69 (96%) were in the public sector, which aligns with the national digital health strategy and the country’s current state of health facility distribution.

Health facilities were initially classified as either urban or rural for spread and inclusion, based on information from the DPPI at MoHS, working in conjunction with the respective District Health Management Team heads. Health facilities were further classified according to the level of their digital health activity. For this mapping exercise only, health facilities were classified into three groups according to low, medium, or high digital health activity based on having no digital health solution, one or two digital health solutions, and three or more digital health solutions, respectively. We sampled a minimum of 5 health facilities per district, selecting 2 each from urban and rural location in each district, as precategorized. Each district prioritized at least one facility with high digital health activity, followed by at least one health facility with medium activity, and finally one with no activity. Because each district had a minimum of one district hospital, only one district hospital was selected in each district irrespective of their digital health activity. Additional health facilities were selected by repeating this selection technique until the desired number was reached in each district. In a situation for which some categories did not exist (eg, no high-activity digital health facility), the required numbers were filled in with other categories.

### Data Collection and Analysis

Ten study enumerators were recruited, trained, and deployed for this exercise in January 2019. The study enumerators visited assigned health facilities and interviewed the head of the health facilities while observing for infrastructures. The enumerators collected data using mobile forms, which were exported into an Excel spreadsheet. The Excel data were then analyzed with the *pandas* and *matplotlib* Python libraries [15].

## Results

### Internet Connectivity

**Figure 3:**
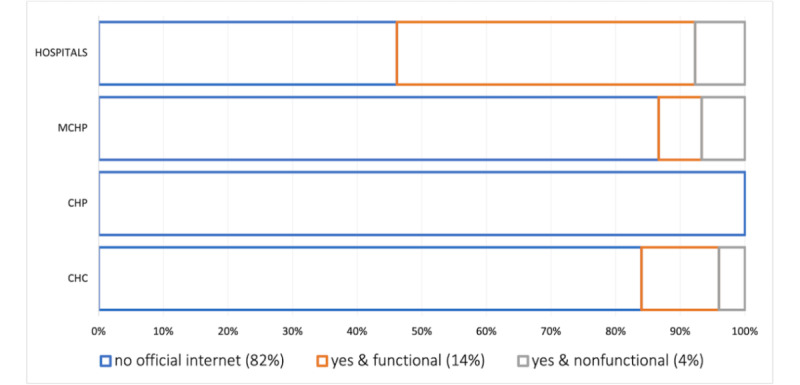
Percentage of health facilities that have institutionally provided internet. MCHP: Maternal and Child Health Post; CHP: Community Health Post; CHC: Community Health Center.

Respondents at 19 of the 72 health facilities surveyed reported having unofficial, private internet access at work, including at 6 of the 13 district hospitals surveyed. Approximately 90% (53/59) of the primary health care facilities surveyed did not have official institutional-provided internet. Likewise, half of the hospitals did not have official internet, as illustrated in Figure 3.

### Electricity

All hospitals surveyed had an electric power source; 6 of them had a national utility grid, 5 had generators, and 1 had solar panels as the primary source. The detailed distribution of the number and type of primary electricity sources according to the different types of health facilities is provided in Table 1. Approximately half of the PHUs surveyed did not have an alternative electricity supply source. All hospitals had one or more alternative electricity supply sources. Approximately half of the PHUs use their primary electricity source (ie, solar) for one purpose only, and the other half use the electricity for all health facility needs.

**Table 1:** Primary electricity sources of the surveyed health facilities (N=72).

Facility type	National utility	Generator	Solar	No electricity	Did not specify
Hospital	6	5	1	0	1
CHC^a^	6	1	17	1	0
CHP^b^	4	0	13	2	0
MCHP^c^	0	0	9	6	0

^a^CHC: Community Health Center.

^b^CHP: Community Health Post.

^c^MCHP: Maternal and Child Health Post.

**Figure 4:**
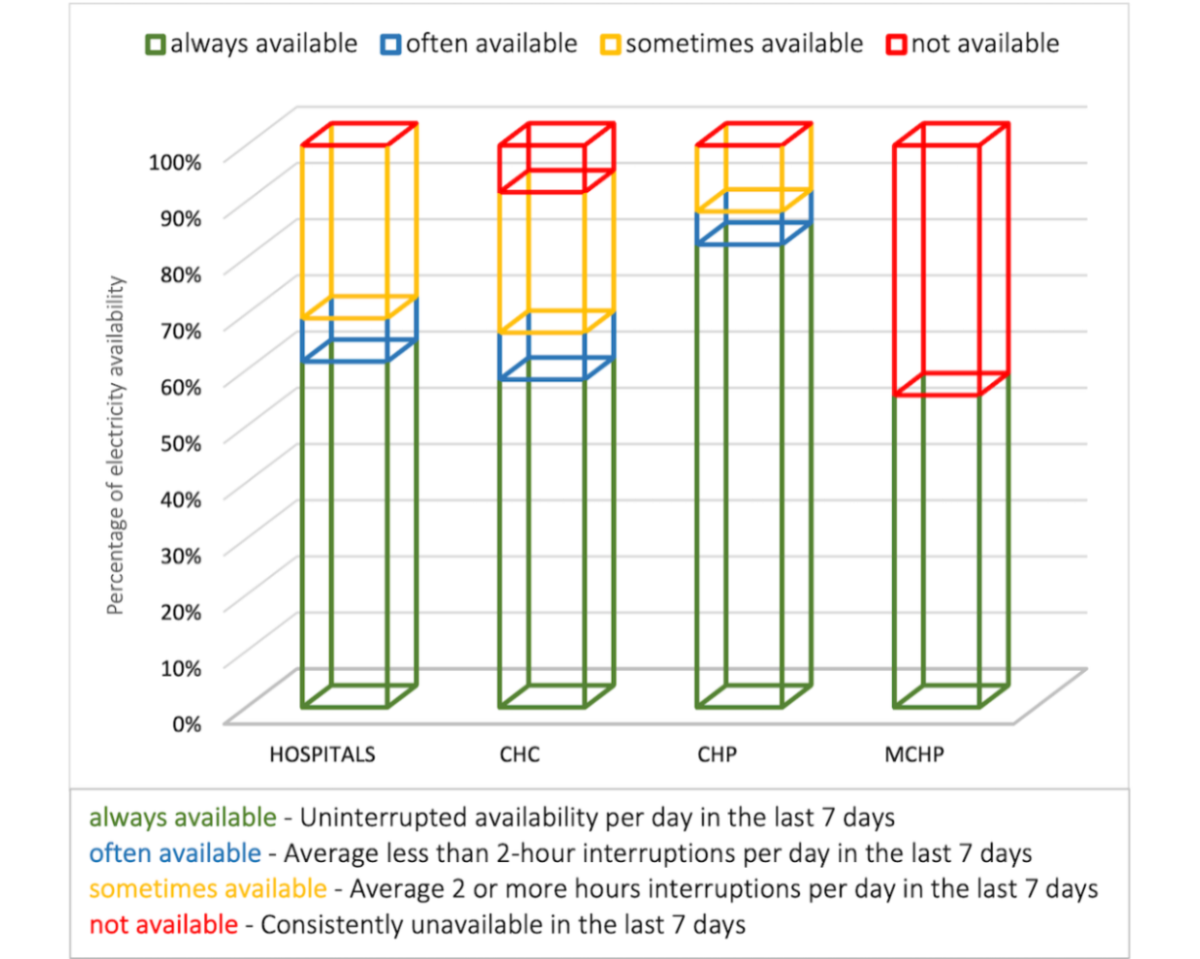
Duration of electricity availability at health facilities. CHC: Community Health Center; CHP: Community Health Post; MCHP: Maternal and Child Health Post.

Respondents at health facilities with primary electricity sources were asked how long electricity was available at their health facilities, using their recall about availability in the 7 days before the survey. Approximately two-thirds of the hospitals surveyed indicated that they had an uninterrupted power supply in the previous 7 days. Moreover, none of the hospitals surveyed reported consistently unavailable electricity. The results for the PHUs were mixed, with the MCHPs having the worst findings (see Figure 4).

Eleven of the 13 hospitals had a generator, and most of the generators at these hospitals were functional. One hospital had a nonfunctional generator and one had no generator. At the time of the survey visit, only 8 of the 11 hospitals with a functional generator had fuel in the event of a power outage. Similarly, only 1 of the 6 PHUs with functional generators had fuel at the survey period. Nine of the PHUs with solar as the primary electricity source had a partially functional solar and inverter system.

### Computing Hardware

All 13 hospitals had at least one functional computer at the time of enumerator visits. Moreover, only 2 PHUs had a functional computer. However, only 7 hospitals had 4 or more functional computers. One hospital had 15 functional computers. Thirty-seven of the health facilities with tablet-based digital health solutions had only one tablet. Other statistics from the survey showed that only 8 PHUs had one smartphone and one hospital had eight smartphones. One PHU had two feature phones and one hospital had one feature phone. Six PHUs had one basic phone (dumbphone) that could only be used for calls and SMS text messaging. Three hospitals had internet modems. One hospital had one modem, one hospital had two modems, and one hospital had three modems.

## Discussion

### Principal Findings

Infrastructure in support of information systems is at the core of the success of an HIS [16]. Individualized care can be better optimized for data use when seamless HIS electricity, computing devices, and internet-network infrastructures are available. This study mapped 72 health facilities, which were first divided into subgroups by health facility type, ownership, and rural-urban distribution. The selection was further stratified to consider digital services and applications use in the health facility for adequate representation. This sample gave a 95% confidence level with an 11% margin of error. This means that the findings in this report are statistically generalizable. This study focused on Sierra Leone, which is considered an excellent example of a low- and middle-income country (LMIC), although we are aware that other LMICs may vary slightly in their characteristics [17].

The trends from our findings show the increasing use of solar solutions for PHUs, which are located mostly in rural locations. These rural facilities are often disconnected from the national electricity utility grid. This is a crucial lesson Sierra Leone shares with other LMICs with similar infrastructure deficits [18]. Some MCHPs still do not have any electricity source, and any health facility digitization depends on electricity. Renewable energy sources are bridging these gaps. Our findings show that 66% (39/59) of PHUs use solar as their primary electric energy source and 15% (9/59) have had no electricity source. This means that over 80% (48/59) of PHUs do not use the national utility as a primary electricity source. This mapping shows without a doubt that direct current–based renewable-energy alternatives may be better suited for targeting off-grid PHUs [19] (see [20] for technical differences between direct and alternating current electricity systems).

The internet distribution analysis showed that 82% (53/59) of all health facilities do not have official institutional-provided internet. However, 26% (19/72) had private internet across health facility types, as shown in Table 2. Three mobile telecommunications service providers (Africell, Orange, and SierraTel) provided internet in all health facility visits. Alternative internet networks such as fiber internet provided by the Ministry of Information [21] and satellite-based internet sources such as SpaceX can better serve off-the-grid health facilities [22]. In the event of no internet, an offline-first HIS solution will be most appropriate.

**Table 2:** Internet infrastructure based on the health facilities survey (N=72).

Health facility	No private internet, n	Has private internet, n
CHC^a^	19	6
MCHP^b^	14	1
CHP^c^	13	6
Hospital	7	6
Total	53	19

^a^CHC: Community Health Center.

^b^MCHP: Maternal and Child Health Post.

^c^CHP: Community Health Post.

**Figure 5:**
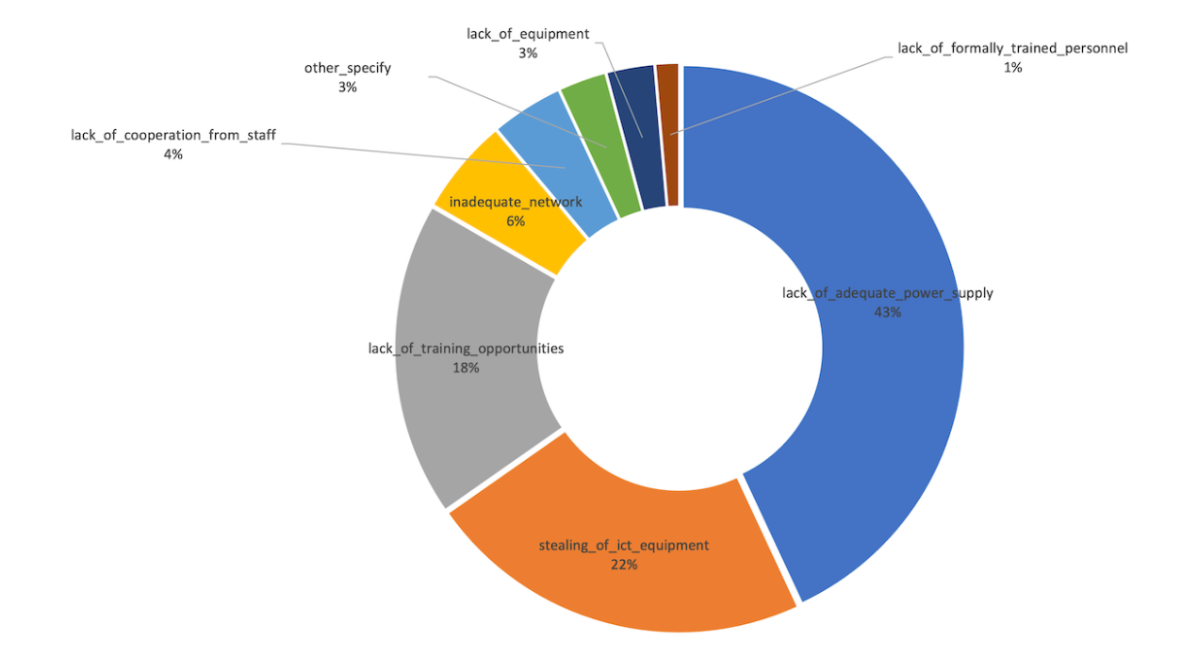
The biggest threats to information communication technology efforts at the health facilities surveyed. ict: information and communications technology.

The majority of the PHUs surveyed had one tablet. Depending on the solutions deployed in these health facilities, one tablet per health facility may or may not be adequate [23]. Some advanced solutions may also not work on tablets, given that only 2 of the 59 PHUs had one computer each. Therefore, software solutions targeting these PHUs should be designed to be tablet-compliant. By contrast, hospitals did not all have the same number of computers. One hospital had 15 functional computers, another had 9, and six hospitals had 4 or fewer functional computers. This shows that computing devices across the hospitals are not evenly distributed, indicating a significant computing infrastructure gap. Among the staff interviewed at the surveyed health facilities, 43% considered that lack of an adequate power supply was the biggest threat to digitization, as shown in Figure 5. Low ICT capacity and theft of digital devices were considered an equally significant threat to digitization in Sierra Leone.

### Limitations

A key limitation of this survey is that the responses were self-reported, although we attempted mitigating against possible bias by ensuring that the enumerators sighted the infrastructure. However, some enumerators were not able to sight the infrastructure for several reasons.

### Conclusion

In this study, we explored the state of infrastructure enabling the digital health environment in Sierra Leone. We surveyed primary and secondary electricity sources, the type and nature of computing hardware, and the internet and connectivity available at these health facilities. We aggregated how often these health facilities have electricity to help determine if a health facility information system can be viable. Disconnected PHUs or hospitals can use alternative electricity sources, fiber or satellite internet, and tablet hardware. This research will support the government in implementing strategies for bridging health facility infrastructure gaps. The next step from this study will be to extrapolate and determine the current infrastructure (electricity, internet, and computing hardware) costs from the national digital health cost plan [9].

## Abbreviations

CHC: Community Health Center
CHP: Community Health Post
DPPI: Directorate of Planning, Policy, and Information
HIS: health information system
ICT: information and communication technology
LMIC: low- and middle-income country
MCH: Maternal and Child Health
MCHP: Maternal and Child Health Post
MoHS: Ministry of Health and Sanitation
PHU: Primary Health Care Unit
WHO: World Health Organization
